# The effect of ear acupressure (auriculotherapy) on sexual function of lactating women: protocol of a randomized sham controlled trial

**DOI:** 10.1186/s13063-020-04663-x

**Published:** 2020-08-20

**Authors:** Sanaz Barghamadi, Zainab Alimoardi, Terry Oleson, Nasim Bahrami

**Affiliations:** 1 Student Research Committee, Qazvin University of Medical Sciences, Qazvin, Iran; 2 Social Determinants of Health Research Center, Research Institute for Prevention of Non-Communicable Diseases, Qazvin University of Medical Sciences, Shahid Bahonar Blvd, Qazvin, Iran; 3 Emperor’s College of Traditional Oriental Medicine, Santa Monica, CA USA

**Keywords:** Ear acupressure, Sexual function, Lactation

## Abstract

**Background:**

Lactation has a negative effect on female sexual function. Hormonal changes during lactation cause changes which might lead to dyspareunia, lack of libido, and anorgasmia. There are various pharmacological and non-pharmacological approaches to treat sexual dysfunction. While pharmacological treatment has multiple unwanted side effects, non-pharmacological therapies such as complementary medicine are a potential safer alternative. The aim of this study is to evaluate the effect of ear acupressure on sexual function of lactating women.

**Methods/design:**

This is a randomized clinical trial with a parallel sham control group. In this study, 76 lactating women between 6 months and 1 year after childbirth were referred to health care centers in Qazvin City and would be invited to participate. Participants will be divided into intervention (*n* = 38) and control (*n* = 38) groups using simple block randomization. Both intervention and sham control groups will be visited over 10 sessions within a 4-day interval. At each visit, the adhesives containing *Vaccaria* seed will be adhered for the intervention group, while non-latex-based adhesives with no *Vaccaria* seeds will be placed on the same ear acupoints for the sham control group. Selected ear acupoints include genitalia (two ear points), pelvic point, master shoulder, and posterior pituitary gland. The women will be asked to hold the seeds on their ears for 3 days and press each ear point three times a day for 20 s. After 3 days, they will be asked to remove the seeds from their ears and rest for 1 day. Sexual function as primary outcome in both groups will be assessed using the Female Sexual Function Index before and immediately after 1 and 2 months after the intervention. Also, Sexual Quality of Life as secondary outcome will be assessed using Sexual Quality of Life-Female (SQOL-F) before and 2 months after intervention. Data will be analyzed using repeated measure ANOVA at the significant level of 0.05.

**Discussion:**

This study is expected to support the impact of ear channel ear acupressure on sexual function in lactating women.

**Trial registration:**

Iranian Clinical Trial Registration Center IRCT20190626044028N1. Registered on 16 August 2019

## Background

The post-childbearing period is a crucial stage in the woman’s life that involves physical, hormonal, mental, social, and cultural changes [1]. Fatigue, insomnia, some stressors such as child care, and changes in the body image along with hormonal changes can reduce interest in sexual life and decrease the number of sexual intercourses during this period [1]. Endocrine function of lactation has a negative effect on sexual function, because prolactin decreases sexual hormones such as androgen and estrogen. This mechanism reduces sexual function because of vaginal dryness, vaginal epithelium atrophy, and dyspareunia [2]. About two thirds of women during lactating period experience at least one sexual problem, such as decreased libido, lack of sexual pleasure, dyspareunia, and vaginal dryness, which usually are resolved within 1 year after childbirth [3, 4]. The prevalence of sexual dysfunction increases from 19 to 63% in the prepartum period [5] to 34 to 91% in the postpartum period [6]. The results of various Iranian studies confirmed the high prevalence (31.5 to 85.4%) of sexual dysfunction during lactation [7, 8]. Therefore, sexual dysfunction is a common problem during lactation that needs more attention from health care providers.

In the post-childbearing period, most women try to return to their sexual life within 8 to 12 weeks after childbirth. However, it may take up to 1 year to resume sexual relationships with the same quality of before pregnancy [9]. Psychotherapy and pharmacotherapy are two main methods for treating female sexual dysfunction (FSD) [10]. However, the US Food and Drug Administration (FDA) does not recommend any foods or medicines for the treatment of FSD [11]. Therefore, non-pharmacological therapies such as complementary medicine are a potentially safer alternative to pharmacological therapy. Acupressure is one of the non-invasive complementary methods of traditional Chinese medicine (TCM) based on the principles of acupuncture [12]. Acupuncture stimulates medical points and meridians that are distributed throughout the body to regulate physiologic reactions [13]. Acupressure, like acupuncture, modulates a person’s vital energy by stimulating acupoints and meridians that are distributed throughout the body [14].

The external ear is one of the several somatotopic microsystems that can be used in acupressure. Ear acupressure, also known as auriculotherapy or auricular acupressure [15, 16], was first presented in ancient Chinese medicine (300 to 500 years BC), but Dr. Paul Nogier was the first Western physician who put forward auriculotherapy in a scientific way [13, 17, 18]. According to this theory, each part of the body is associated with a specific part of the ear that reflects the physiological or pathological state of the body [13]. In this method, the outer surface of the ear (auricle) can be stimulated to reduce pathological conditions in other parts of the body [13]. Ear stimulation can be performed in various ways such as manual finger pressure, electrical stimulation, lasers, different types of needles, magnetic seeds, and seeds to strengthen neural connections [18]. The World Health Organization (WHO) has recognized ear acupuncture as a promising therapeutic approach, because of its efficacy in managing health disorders [15]. Ear acupuncture has been recognized as a form of acupuncture that can affect all body organs [19]. Ear acupressure is an easy, non-invasive, and safe therapy [20, 21]. It is easy to use for mothers who need to take care of their child in the post-childbearing period [13].

There are several studies supporting the effects of ear acupressure on insomnia [20, 22], menstrual cramps and dysmenorrhea [21, 23, 24], premenstrual pain [25], chemotherapy-induced nausea and vomiting [26], smoking cessation [27], obesity [18], and shortness of breath [28]. However, the effect of ear acupressure on sexual function has not been studied.

### Research aim

This study is designed to investigate the effect of ear acupressure on the sexual function of lactating women.

## Methods

### Design and setting

**Fig. 1:**
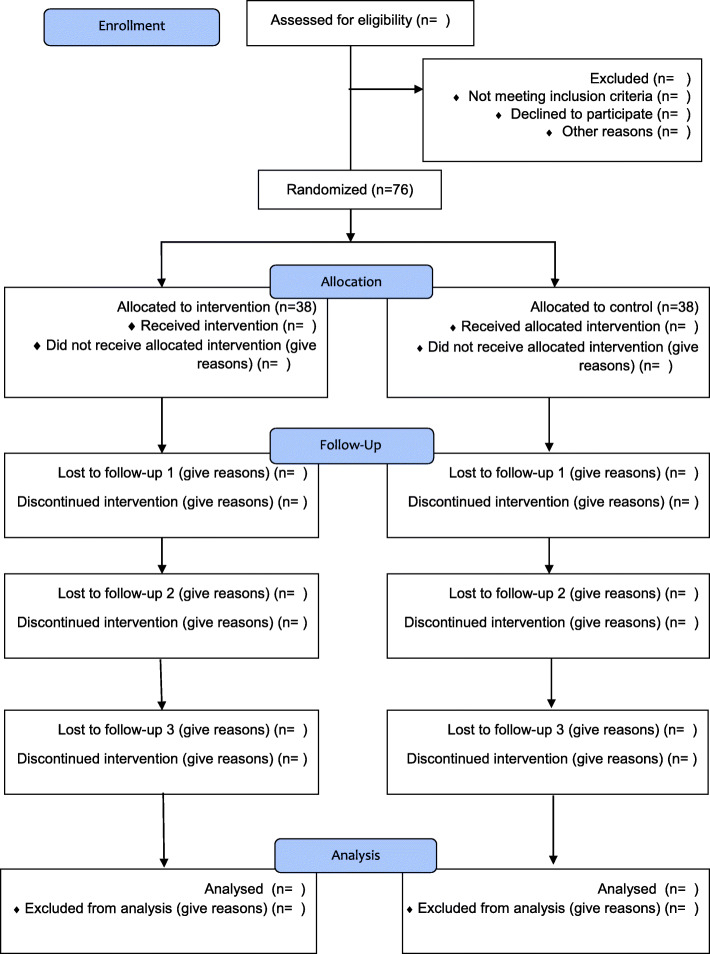
The CONSORT flow diagram

This is a randomized clinical trial with a parallel sham controlled group. The study is designed in accordance with the CONSORT standards. Lactating women referred to health care centers in Qazvin City, Iran, will be invited to participate. Figure 1 shows the CONSORT flow diagram. The current protocol is organized based on the SPIRIT-TCM extension [29].

### Participants

In this study, breastfeeding women between 6 months and 1 year after childbirth will be referred to health care centers in Qazvin City, Iran, and they will be invited to participate in the study. Inclusion criteria will be willingness to participate in the study, being literate, being primiparous, breastfeeding, full-term singleton delivery, lesion-free auricle, no ulcer and pain in the ear, and ability to present for all intervention sessions. Exclusion criteria will be being away from their spouse for more than 1 month, having complications during pregnancy, or having postpartum depression in the post-childbearing period. These criteria were diagnosed using the Edinburgh Postpartum Depression Scale, substance abuse in the individual or the spouse, and history of illnesses affecting sexual function in a woman or her spouse (e.g., premature ejaculation, cardiovascular, mental health disorders, thyroid diseases, any form of cancer, or injuries of the genital area). The use of drugs affecting sexual function such as psychotropic, cardiovascular, neurological, and hormonal drugs will also be excluded. After the initial screening, the Demographic and Obstetric Questionnaire and Female Sexual Function Index (FSFI) will be completed by the participants before any intervention.

### Role and qualification of practitioners

The research team consists of one specialist in auriculotherapy (TO), two specialists in reproductive health (ZA and NB), and one midwifery postgraduate student. The protocol of intervention was designed based on the literature review and expert opinion of TO who is an expert in auriculotherapy and is a journal editor for several international journals. Other team members have been qualified in this technique before designing present research. Participant screening and care providing will be done by SB, and ZA and NB will monitor her during the first 10 sessions to ensure the fidelity of providing intervention.

### Sample size calculation

Sample size is estimated according to the study of Bokaie et al. with the mean and standard deviation of total sexual function score has been reported as 24.65 ± 3.47 and 22.27 ± 3.03 in the intervention (telephone counseling group) and control groups, respectively [30]. Therefore, considering the first type error (α) = 0.05 (95% confidence), the second type error (β) = 0.2 (80% power) and error *d* = 5, and the possibility of 10% loss to follow-up, 38 individuals for each group are required. So, the total sample size will be 76 people.

### Intervention

#### Ear acupressure group

**Fig. 2:**
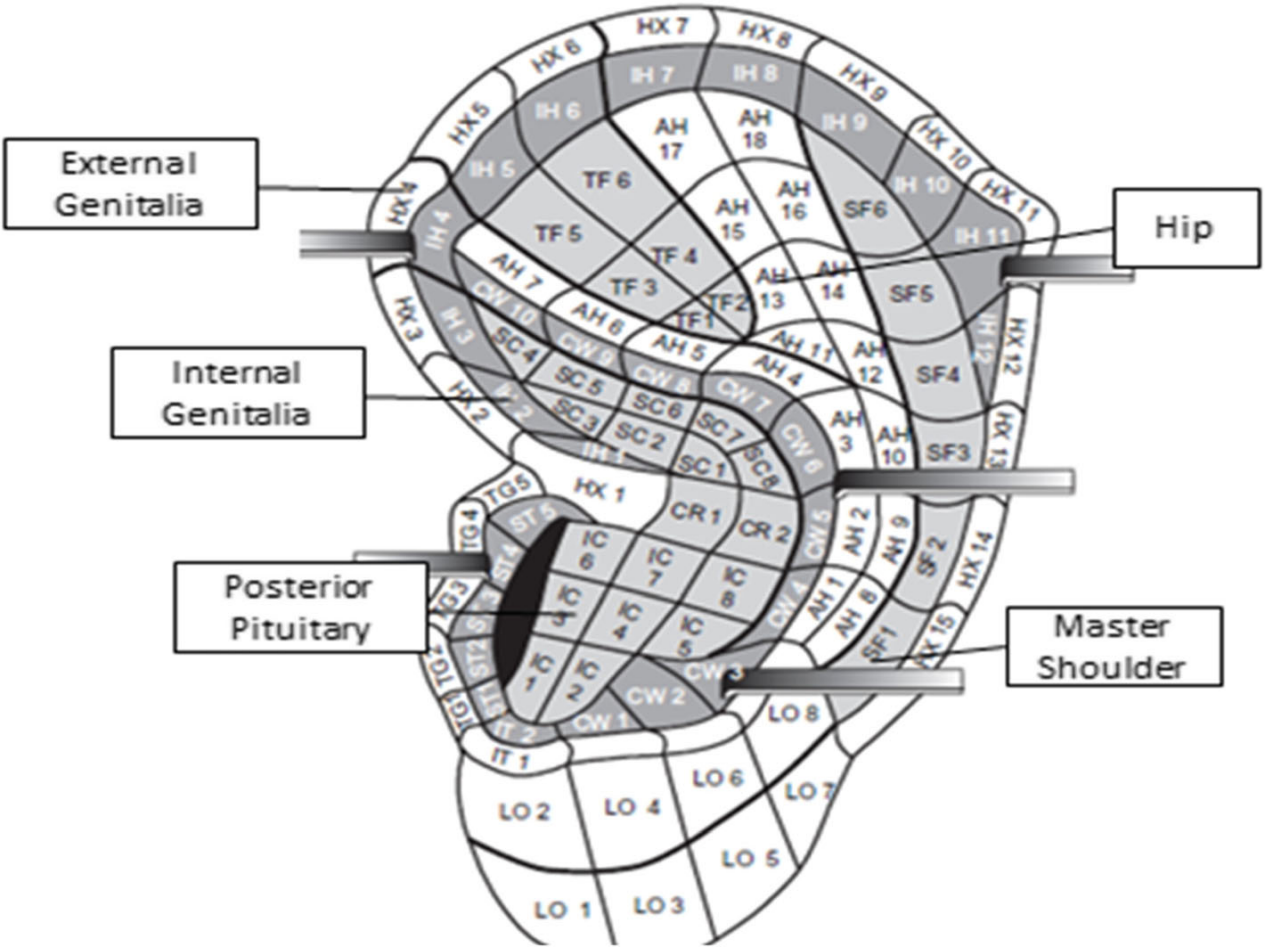
The selected ear acupoints

The researcher will clean the auricle using 75% alcohol and then place *Vaccaria* seeds using non-latex-based adhesives on ear acupoints for genitalia, pelvis, shoulder, and the posterior pituitary gland. Figure 2 illustrates the intended ear acupoints. Intervention is designed for 10 sessions within a 4-day interval. During these sessions, the seeds are placed on the designated ear acupressure points. The women will be instructed to keep the seeds on their ears for 3 days, during which each point should be compressed three times a day for 20 s. The compression should be performed with moderate stimulation through pressing steadily and slightly tighter until feeling a slight tingling and discomfort. After 3 days, they will be asked to remove the seeds from their ears and rest for a day [29]. The women will be reminded daily by sending the text and will be reminded by phone for the next interventional session. This procedure will be performed for ten sessions for each participant. It should be noted that if a participant has problems during the use of the method for any reason (displacement of seeds, discomfort, etc.), she can remove the previous seed and ask the researcher to re-attach new seeds.

#### Sham control group

The control group will have sessions that will be the same as the intervention group except that no *Vaccaria* seeds will be placed on the ear acupoints.

### Measures

In this study, four measures will be used for data collection.

#### Demographic and Obstetric Questionnaire

The demographic section includes questions such as age, education level, occupation, place of residence, economic status, age at marriage, and length of marriage. The obstetric section includes questions regarding the number of pregnancies, type of contraceptive method, delivery date, type of delivery, perineal injury, instrumental delivery, history of painful intercourse in prenatal period, infant gender, birth weight, first lactation, number of intercourse per month before pregnancy, onset of first intercourse after delivery, and average number of intercourses per week during lactation. Validity of this questionnaire will be confirmed by some of the faculty members of the midwifery department of Qazvin University of Medical Sciences, Iran.

#### Female Sexual Function Index (FSFI)

It has been designed by Rosen et al. to evaluate sexual function in women over the past 4 weeks [31]. It consists of 19 items subdivided into six subcategories of sex desire (2 items), arousal (4 items), lubrication (4 items), orgasm (3 items), satisfaction (3 items), and pain (3 items). These subcategories have response ranges from 0 or 1 to 5, with higher scores indicating better sexual performance [32]. The questionnaire has been used in many studies abroad and has shown to have a high degree of internal consistency, reliability, and validity [33]. The findings of Fakhri and Mohammadi’s study showed that the Farsi version of FSFI, FSFI-IV, is a reliable and valid instrument with appropriate psychometric properties to evaluate women’s sexual function [32, 34]. Reliability of the scale has been calculated through stability analysis or calculation of the internal consistency coefficient. The Cronbach’s alpha coefficient was 0.70 and was higher for all domains and for the whole scale [34]. In addition, reliability was confirmed by the test-retest method and the correlation coefficient was reported high indicating that FSFI-IV is reusable within a 4-week interval [31, 35–38]. The maximum score for each domain is 6 and for the whole scale is 36. Also, any score less than 28 will be used to indicate sexual dysfunction [34].

#### The Edinburgh Postpartum Depression Rating Scale

This 10-item scale has been designed to detect depression from 6 weeks after delivery. The Edinburgh Scale scores vary between 0 and 30 with cutoff point 12 and above to detect postpartum depression [39]. Psychometric evaluation of the Farsi version of this scale is carried out in 2015 [40]. The Cronbach’s alpha for the Edinburgh Scale has been reported as 0.7. Validity of the Edinburgh Beck Scale has been reported as 0.44. The findings of the study confirmed the high validity of the Farsi version of the Edinburgh Scale for the diagnosis of postpartum depression [40].

#### The Sexual Quality of Life-Female (SQOL-F)

It is a short tool that specifically assesses the relationship between sexual function and women’s quality of life. It has been developed by Symonds et al. [41]. This 18-item questionnaire focuses on sexual self-esteem, emotional affairs, and relationships. Each item is responded with a Likert scale of strongly agree (score 6) to strongly disagree (score 1). Higher scores indicate better quality of sexual life for women [41]. Validity and reliability of the Farsi version of this scale have been confirmed by Maasoumi et al. [42].

### Primary outcome

Primary outcome of this study is to investigate changes of sexual function using the FSFI.

### Secondary outcome

The secondary endpoint is investigating the quality of women’s sexual life, which will be assessed using the Sexual Quality of Life Questionnaire. Any harm or side effects will be also reported.

### Study procedure

After obtaining the necessary permissions from the Ethics Committee of Qazvin University of Medical Sciences, registration at the Iranian Clinical Trial Registration Center (IRCT), and obtaining the permission from Qazvin Nursing and Midwifery School, the researcher will attend for recruitment in Qazvin Health Centers. The lactating women of each center will be invited for participation. The process and purpose of the research will be explained to those who meet the inclusion criteria. Also, they will be ensured that their information will be kept confidential and that the questionnaires will be anonymous and coded in accordance with the research ethics. They will enter into the study after signing the written consent form. The researcher will ensure that they can leave the study at any time. After enrolling eligible individuals, a simple block randomization will be used. Participants will be randomly assigned to the intervention and control groups.

**Fig. 3:**
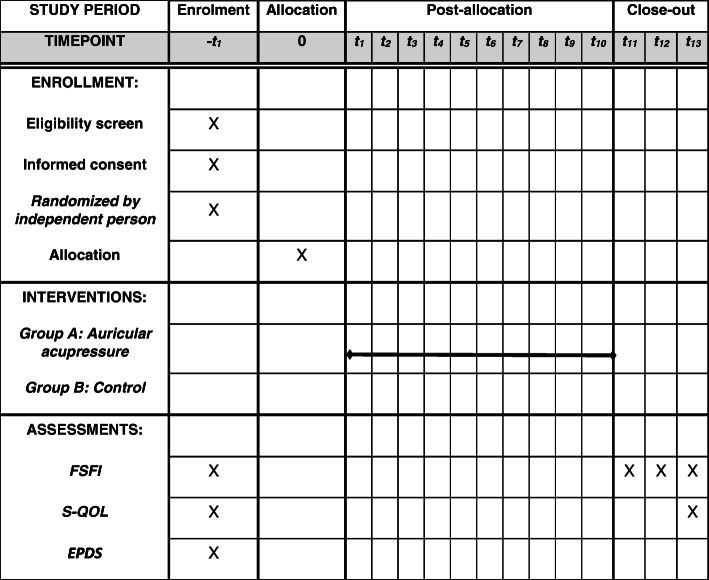
Schedule of enrolment, interventions, and assessments

The questionnaires will be filled out by the participants before the intervention. Then, the intervention (acupressure) will be performed as mentioned above. Evaluation of sexual function in both the intervention and control groups will be performed using the FSFI questionnaire immediately, 1 and 2 months after the intervention. Figure 3 provides the timeline of recruitment, allocation, and assessment.

### Statistical analysis

Data will be analyzed using the chi-square test, Fisher exact test, and independent *t* test via the SPSS v.24 software. The Kolmogorov-Smirnov and Shapiro tests will also be used to check for the normal distribution of sexual function scores. Descriptive statistics (number, mean, and standard deviation) will be used to describe the demographic characteristics of the participants. The repeated measure ANOVA will be used to compare the mean sexual function of women before and after the intervention. The significance level of all tests is set as *p* < 0.05.

### Methods to protect against bias

#### Randomization and allocation

Sampling will be made using a two-step sampling method. In the first step, Qazvin City will be considered as five geographical districts. Next, two health centers will be randomly selected in each of these five districts of Qazvin City. Secondly, women referred to selected health centers will be assessed for eligibility and, in case of willingness to participate in the study, will be recruited. They will be randomly assigned to two groups as follows: One letter is assigned to each group (A: intervention group, B: control group), and all possible conditions for the 4-block will be written and numbered, as follows: 1.AABB 2.ABAB 3.BBAA 4.BABA 5.ABBA 6.BAAB 7.

In a simple block randomization method (using the table of random numbers), numbers of blocks will be selected until the specified sample size is achieved. The random allocation sequence is specified. For example, if the numbers given are 3, 2, 2, 1, and ..., respectively, the allocation sequence will be as follows: AABB ABAB ABAB BBAA. As a result, each participant will have a unique code (*n* = 76).

#### Allocation concealment

For this purpose, after preparing the allocation sequence, the sequence is annotated on paper and placed in opaque sealed envelopes, which will be numbered consecutively. The questionnaires will be coded in the same manner. A questionnaire with the same code will be completed by the person who receives code 1 intervention. The assignment sequence and its concealment will be performed by someone outside the research team.

#### Blinding

Participants, outcome assessor, and statistical analyzer will be blind to which group a participant was placed. The individual entering the data from the participant questionnaires on demographic information, postpartum depression, female sexual functioning, and female quality of life will be blind to which participant was assigned to the auricular acupressure seed group or the sham control group.

#### Treatment fidelity

One of the researchers (SB) is responsible to perform intervention. She has participated specific course on auriculotherapy and is supervised by acupuncturist (MHA) about how to perform the intervention. Approximately 15% of intervention sessions will be run under supervision of the acupuncturist to ensure that all acupoints are chosen correctly.

#### Data management

One of the researchers (SB) will be responsible for collecting data at each study stage and will assign another individual for the task of data entry into the SPSS software. This process will be performed under supervision of NB and ZA.

#### Ethical and safety issues

The research protocol will be reviewed and approved by the Ethics and Human Research Committee of Qazvin University of Medical Sciences (decree code: IR.QUMS.REC.1398.056). It is registered on the Iranian Clinical Trial Registration Center under decree code of IRCT20190626044028N1. In addition, the following ethical considerations will be considered throughout this research: obtaining written informed consent, the volunteer nature of participation in the study, ability to withdraw from the study at any time, and confidentiality of information even during the publication of findings. All ethical issues related to clinical trials will be considered. Informed consent during recruitment phase will be obtained by SB.

## Discussion

To the best of our knowledge, this study is the first randomized clinical trial investigating the effect of ear acupressure on the sexual function of lactating women. The strength of this study is the randomized design with parallel treatment procedures and a large sample size, but due to the importance of the treatment practitioner knowing whether they are placing an adhesive patch with a *Vaccaria* seed or without one, a true double blind study is not possible. Acupressure has no known side effects, is non-invasive, and is easy to use [20, 21]. Therefore, the therapist and clients can develop a sense of closeness, trust, and confidence [20]. Several studies have shown promising results about the effect of ear acupressure on insomnia [20, 22], menstrual cramps and dysmenorrhea [21, 23, 24], premenstrual pain [25], chemotherapy-induced nausea and vomiting [26], smoking cessation [27], obesity [18], and shortness of breath [28]. In the study of Oakley et al., acupuncture was affective in premenopausal women with sexual function [43]. In line with previous literature, this study would provide insights about the impact of ear acupressure on sexual function in lactating women.

## Data Availability

Analyzed data and materials will be de-identified and published.

## Abbreviations

CONSORT: Consolidated Standards of Reporting Trials
SQOL-F: Sexual Quality of Life-Female
